# PER3 variable number tandem repeat (VNTR) polymorphism modulates the circadian variation of the descending pain modulatory system in healthy subjects

**DOI:** 10.1038/s41598-019-45527-y

**Published:** 2019-06-27

**Authors:** Fabiana Carvalho, Mario Pedrazzoli, Assunta Gasparin, Franciele dos Santos, Maxciel Zortea, Andressa Souza, Iraci da Silva Lucena Torres, Felipe Fregni, Wolnei Caumo

**Affiliations:** 1 Post-Graduation Program in Medicine: Medical Sciences, School of Medicine, Universidade Federal do Rio Grande do Sul (UFRGS), Porto Alegre, Brazil; 2 Laboratory of Pain & Neuromodulation at Hospital de Clínicas de Porto Alegre (HCPA), Porto Alegre, Brazil; 3 School of Arts, Science, and Humanities, Universidade de São Paulo, São Paulo, Brazil; 4 School of Medicine, UFRGS, Porto Alegre, Brazil; 5 Postgraduate Program in Health and Human Development, La Salle Universitary Center, Canoas, RS Brazil; 6 Pharmacology Department, Instituto de Ciências Básicas da Saúde, UFRGS, Porto Alegre, Brazil; 7 Physical Medicine & Rehabilitation Department, Center of Neuromodulation & Center for Clinical Research Learning, Spaulding Rehabilitation Hospital, Harvard Medical School, Boston, Massachusetts USA; 8 Pain and Palliative Care Service at HCPA, Porto Alegre, Brazil; 9 Department of Surgery, School of Medicine, UFRGS, Porto Alegre, Brazil

**Keywords:** Biomarkers, Pain, Genetics research

## Abstract

We evaluated the circadian pattern of variation of the descending pain modulatory system (DPMS) using a conditioned pain modulation (CPM) paradigm according to the variable-number tandem-repeat (VNTR) of the clock gene PER3 polymorphism. We assessed the relationship between the genotypes PER3^4/4^ and PER3^5/5^ and the temporal pattern of variation across the day using the following measures: the heat pain threshold (HPT), the cold pressure test (CPT), and the serum levels of BDNF and S100-B protein. The ∆-values (from afternoon to morning) of these measures were used for the analysis. The circadian phenotype was according to the mid-point sleep time established by the Munich ChronoType Questionnaire (MCTQ). We included 18 healthy volunteers (15 women) ages 18 to 30. A Generalized Linear Model (GLM) revealed a significant difference in the ∆-CPM-task between Per3^4/4^ and Per3^5/5^ genotypes, with means (SDs) of −0.41 (0.78) vs. 0.67 (0.90) (χ^2^ = 7.256; df = 1′ P = 0.007), respectively. Both sleep deprivation of at least 2 h/day (B = −0.96, 95% confidence interval (CI) = −1.86 to −0.11)) and the ∆-S100-B protein (−0.03, 95% CI = −0.06 to −0.02) were negatively correlated with the ∆-CPM-task, while the ∆-BDNF was positively correlated with the ∆-CPM-task (0.015, 95% CI = 0.01 to 0.03). We observed a difference in the ∆-CPT between PER3^4/4^ and PER3^5/5^ (0.11 (4.51) vs. 4.00 (2.60), respectively) (χ^2^ = 22.251; df = 1 P = 0.001). These findings suggest that the polymorphism of PER3^5/5^ is associated with a decrease in the inhibitory function of the DPMS over the course of the day. However, sleep deprivation is an independent factor that also reduces the inhibitory function of the DPMS, regardless of the PER3 VNTR polymorphism.

## Introduction

The primary circadian pacemaker is located in the suprachiasmatic nucleus (SCN). It controls many physiological and behavioral variables through synchronized clock-gene functions that control rhythms in the central and peripheral nervous system^1,2^. Although the PERIOD3 (PER3) clock gene plays a limited role in the primary clock mechanism, it does drive circadian rhythms in humans according to a variable number tandem repeat consisting of either 4 or 5 repeated 54-bp sequences encoding 18 amino acids (PER3^4^ or PER3^5^). This repeat may affect chronotype, either via direct effects on the clock or through other mechanisms such as sleep homeostasis^3,4^. The PER3 polymorphism has been associated with circadian typology, where PER3^5/5^ is linked to a morning-type circadian typology characterized by advanced phase of entrainment^4–6^, whereas PER3^4/4^ is typically linked to an evening-type typology that exhibits delayed phase of entrainment and a higher propensity towards sleep deprivation^7^. Also, an earlier study found that individuals with familial advanced sleep phase disorder are prone to depression^8^. Also, individuals with PER3^4/4^ are likely predisposed to higher levels of anxiety^9^.

The effects of PER3 variants on mood are mediated by the ongoing reduced sleep deprivation coupled with deregulated internal circadian functions, such as body core temperature and levels of melatonin and cortisol^10^. Sleep deprivation also eliminates distraction-based analgesia^11^, decreases the pain inhibition by the diffuse noxious inhibitory controls in humans^12,13^ and stress-induced hyperalgesia in rodents^14^. In humans, the biological processes of sleep, circadian rhythmicity, and pain interact to explain how pain sensitivity may vary across the 24 h day, with the highest sensitivity occurring during the evening^15^. Chronic pain frequently co-occurs with depression, poor sleep quality, and anxiety^16–18^. If PER3 variants linked to delayed phase shifts affect mood and sleep quality, it would appear likely that individuals that have these PER3 variants would tend to have greater sensitivity with respect to pain perception^7,9,19^. Pain exhibits circadian variation regardless of whether pain responses are measured subjectively or objectively^15^. In the same way, the top-down inhibition of pain processing in the dorsal horn exhibits a rhythm across the day^20^. Indeed, all of these factors could influence the inhibitory function of the descending pain-modulating system (DPMS), which has a central role in the pathophysiology of chronic pain. This system is impaired in chronic pain of different causes (i.e., fibromyalgia, osteoarthritis, rheumatoid arthritis, neuropathic pain, etc.). Additionally, its poor function has been a predictor for postoperative pain^21,22^.

Previously, we found that the disengagement of the DPMS is correlated with changes in serum brain-derived neurotrophic factor (BDNF)^23^. BDNF is a prime mediator of activity-dependent neural plasticity. This neurotrophic factor is synthesized in both the central and peripheral nervous system and by astrocytes^24^. BDNF and the S100-β protein are both key neuroglia mediators that have been inversely correlated with the pain pressure threshold^25^. BDNF is tightly associated with a higher score in the Central Sensitization Inventories^26^ as well more substantial dysfunction of the DPMS^23^. According to pre-clinical and clinical studies, sleep deprivation appears to increase pain by decreasing the inhibitory activities of the DPMS and by increasing descending pain facilitatory activity^12,27^. The number of studies about the circadian pattern of pain is growing; however, a gap persists in this field with respect to comprehending the inhibitory function of the DPMS according to different clock gene polymorphisms, nominally PER3, that alter the length of the circadian period. In addition, there is a poor understanding of how these polymorphisms can modulate the relationship between the DPSM and changes in the neuroplasticity processes across the day as measured by neuroplasticity markers (BDNF and S100-β protein). Thus, we conducted this exploratory study to assess whether the circadian pattern of variation in the functioning of the DPMS, measured by change in the Numerical Pain Scale (NPS0-10), differed between and within groups of subjects according to PER3 polymorphic variants or PER3^4/4^ and PER3^5/5^ genotypes (primary outcome). Secondary outcomes were the circadian variations in the pain measures (heat pain threshold (HPT), cold pressure test (CPT), and serum levels of BDNF and S100-B protein). We also considered sleep deprivation in all analyses.

## Materials and Methods

### Design

We conducted an exploratory cross-sectional study. The sections were reported according to STROBE guidelines^28^. This study was conducted according to international ethical standards based on the Declaration of Helsinki. The Institutional Board Ethics Committee at the Hospital de Clínicas de Porto Alegre (HCPA) approved the protocol of this study (Applications No. 00614812.4.2003.5327 and 13-0455). All participants gave their oral and written informed consent.

### Participants

**Figure 1:**
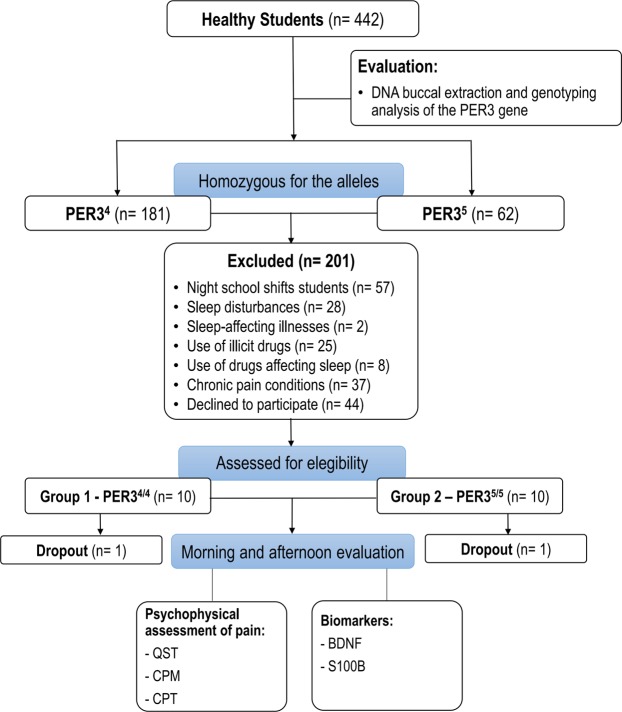
Flow of study.

Participant selection was conducted in two phases. In phase 1, we selected 442 participants who filled out the Munich ChronoType Questionnaire (MCTQ). DNA buccal samples were collected for analysis of genotypes of PER3 alleles. Inclusion criteria was to be homozygous for the PER3^4/4^ and PER3^5/5^ alleles, to be university students who study during the morning and afternoon and to have between 18 and 30 years old, which reduced considerably the number of eligible participants. In addition, the exclusion criteria were students with night school shifts, those who reported sleep disturbances or sleep-affecting illnesses, those who were currently using illicit drugs or drugs that affect sleep, and those who presented chronic pain conditions. In phase 2, a subsample of 20 undergraduate students was selected. They were carefully screened via a standardized questionnaire to ensure that they were healthy, free of medicines and drugs, and had no history of psychiatric disorders. Details of the study can be seen in Fig. 1.

### Instruments and assessments

The instruments used to assess psychological state were previously validated in the Brazilian population^29,30^. Two independent examiners who were blinded to the aim of the study conducted the protocol and assessments. They were trained to apply all tests and to obtain the pain measures. At baseline, the following instruments were employed: the Beck Depression Inventory (BDI II)^30^, the Pittsburgh Sleep Quality Index to assess sleep quality^31^, and a short State-Trait Anxiety Inventory (STAI)-Form X, with scales obtained using the Rasch model^29^. The state and trait scores ranged from 13 to 52 and 12 to 36, respectively. For the assessment of demographic data and medical comorbidities, a standardized questionnaire was used.

### Outcomes and factors

The primary outcome was the DPMS measured using the NPS (0–10) during the CPM-Task, as assessed by the delta value (∆)-CPM (i.e., change in the measures from afternoon to morning). The secondary outcomes were the changes as measured by the ∆-values (from afternoon to morning) in the cold pressure test (CPT) and the heat pain threshold (HPT). The primary factor of interest was the PER3^4/4^ and PER3^5/5^ genotypes of the PER3 VNTR polymorphism. Another factor of interest was the change in the neuroplasticity markers as assessed by the ∆-value (from afternoon to morning) of serum BDNF and S100-β (described further).

### Pain measures

In this study, we evaluated pain as a response to a standardized nociceptive stimulus using Quantitative Sensory Testing (QST), including the Conditioned Pain Modulation task (CPM-task) and the HPT. The participants remained seated, and a thermode (30 × 30 mm) was positioned on the forearm of the dominant side of the body.

For HPT, to perform QST we used a computerized version of the thermostat (Heat Pain Stimulator 1.1.10, Brazil)^32^ to determine the HPT. The temperature started at 32 °C, and the thermode was heated at a rate of 1.0 °C/sec to a maximum of 52 °C, when the temperature began to drop. For the HPT, the participants were asked to press a button when they “felt the first pain sensation”. HPT was determined by the average of three evaluations with a 40 s interval between them.

**Figure 2:**
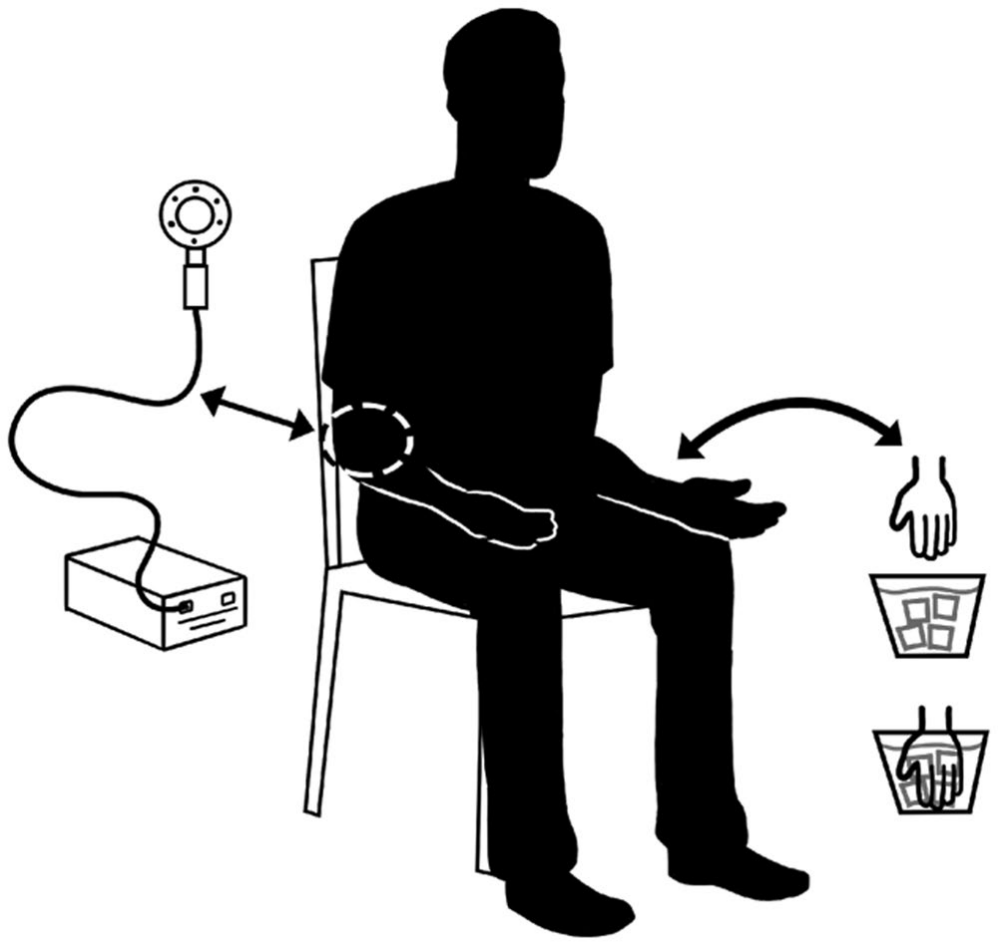
CPM-task and QST.

For the CPM score, we evaluated the pain intensities evoked by two tonic heat stimuli, one before and one during the CPM-task. We applied to the dominant hand a QST heat pain stimulus (test stimulus) of an intensity that elicited a pain score of 6/10 on the NPS (0–10) before the CPM-task (time T0). During the CPM-task (T1), a tonic stimulus (conditioning stimulus) was used and consisted of immersion of the non-dominant hand in cold water at a temperature of 0–1 °C for 30 seconds. A water-proof thermometer was used to confirm this temperature. The heat pain stimulus (test stimulus) with QST procedure was applied a second time for 30 seconds simultaneously with the conditioning stimulus (cold water), and the participant had to rate the pain perceive for the test stimulus again, on a NPS (0–10) scale. It is expected a reduction in pain intensity perception in T1 as compared to T0. The larger this reduction, the stronger the inhibitory function of the DPMS. Thus, CPM score consists of the score on the NPS 0–10 test stimulus during T1 (QST + CPM) minus the score at T0 (6/10). Figure 2 illustrates the CPM-task test.

Finally, for the Cold Pressure Test (CPT), the non-dominant hand was immersed in ice water (0 to 1 °C) for a maximum of 120 s. The outcome was the maximum tolerance for the pain of maintaining the non-dominant hand in the ice water, measured in seconds. Subjects were recorded as 120 seconds if they did not withdraw their hand for the maximum time.

### Genotyping measures

For genotyping, tissue samples of the oral mucosa were taken for DNA extraction using a sterile swab. The swabs were frozen (−80 °C) after the collection until DNA extraction. DNA was extracted from the oral mucosa cells according to standard methods. In the PER3 gene, concerning the VNTR polymorphism, since the difference in the number of base pairs between the two alleles was reasonably large (54 bp), genotyping could be done by PCR alone and visualized in 1% agarose gel. The primers used for amplification of the region of interest and the conditions of the reactions were the same as described by Archer *et al*.^5^.

### Chronotype measures

Chronotype was assessed using the Munich ChronoType Questionnaire (MCTQ)^33^, which consists of one general and nine specific (relative to work or free days) questions aiming to evaluate chronotype through self-reported individual sleeping phases. The MCTQ asks the following about work and work-free days: when people go to bed, how long they need to fall asleep and when they wake up how long they need to get up^34^. The MCTQ established the chronotype and social jetlag. The MCTQ assesses the chronotype as a phase of entrainment (the difference between the period of circadian rhythm and that of the zeitgeber). The chronotype (MSFsc) is calculated as the midpoint between sleep onset and sleep offset on work-free days, corrected for oversleeping if individuals sleep longer on work-free days than on work days. We included in this study individuals with MSFsc values that were earlier than the first or later than the third quartile, that is, those classified as being of morning and evening typology, respectively, according to the MSFsc. Social jetlag represents the misalignment of biological and social time^35,36^. Social jetlag also measures sleep deprivation, defined by the difference between sleep onset on workdays and on free days. We defined differences of 120 minutes or greater as sleep deprivation.

### BDNF and S100-β measurement

We also measured serum levels of brain-derived neurotrophic factor (BDNF) and S100-β protein to obtain neuroplasticity and inflammatory indices. For the determination of the biological markers, 20 mL of blood was collected from each participant in the morning and the afternoon before the evaluations. Blood samples were centrifuged in plastic tubes at 4500 rpm for 10 min at 4 °C, then stored at −80 °C. Serum BDNF was analyzed using the Enzyme-Linked Immunosorbent Assay (ELISA) using monoclonal antibodies specific for BDNF (R&D Systems, Minneapolis, United States #DY248, BDNF lowest detection limit = 11.7 pg/mL). Serum S100-β was determined using the Human S100-β ELISA Kit (Chemicon/Millipore, Billerica, MA, USA #EZHS100B-33K, S100-β lowest detection limit = 2.74 pg/mL).

### Statistical analyses

Descriptive statistics were used to summarize the main socio-demographic features of the sample. For the comparisons between groups for categorical variables, the chi-squared and Fisher’s exact tests were used. To compare continuous variables, the t-test for independent samples and the Wilcoxon Mann-Whitney test were used. The normality of variables was tested by the Shapiro-Wilk test. To examine differences according to temporal variation within groups, the Wilcoxon Mann-Whitney test was used for the dependent sample. To perform the analysis, we calculated the ∆-value (from afternoon to morning) for all outcomes. Spearman correlation (ρ) analyses were performed to examine relationships between pain measures, chronotypes and biochemical variables. A linear mixed model (LMM) was used to compare the outcomes ∆-CPM (the NPS (0–10) score during the CPM-task measured in the afternoon minus the same score from the morning) and ∆-CPT between *PER3* genotypes (PER3^4/4^ and PER3^5/5^), followed by Bonferroni’s Multiple Comparison Test. The ∆-CPM and ∆-CPT were adjusted for sleep deprivation, ∆ values of S100-β protein and ∆ values of BDNF. For all analyses, we considered an error Type I two-sided (bicaudal) α = 0.05. For the post hoc sample size calculation, the power of this study’s analysis is based on the difference in mean scores on the Numerical Pain Scale (NPS 0–10) during the CPM-task between the PER3^4/4^ and PER3^5/5^ genotypes, which were −0.54 (0.78) and 0.70 (0.90), respectively. This difference of 1.24 between the group means results in a statistical power of 84% (with a 2-tailed α level of 0.05).

### Perspective

These findings showed that the circadian variation of the descending pain modulatory system’s functioning during the conditioned pain modulation task (CPM-task) varied according to Per34/4 and Per35/5 polymorphisms. This factor may explain the intra-individual variability in pain responses throughout the day. Hence, the comprehension of the relationship between clock genes and the pain modulatory system may contribute to improved allocation of therapeutic approaches to acute and chronic pain across the day.

## Results

The general characteristics of the sample and comparative analyses used to check for differences between genotypes of the PER3 polymorphism are presented in Table 1. The groups were similar in all measures. Of the 20 subjects assessed, two were excluded from the data analyses, one from each group, due to their mean NPS 0–10 score during the CPM-task exceeded three times the standard deviation of their respective groups. Final sample was composed of 18 participants. According to the MCTQ, in the PER3^4/4^ group, 81.8% of participants were classified as morning-type and 18.2% as intermediate-type, while PER3^5/5^ was composed of 55.6% morning-type and 44.4% intermediate-type. The prevalence of morning-type in the PER3^4/4^ is statistically higher (χ^2^ = 4.87; *P* = 0.04). Based on the MCTQ, no evening-type individuals were found in the groups.

**Table 1:** Sociodemographic and health characteristics of the sample according to PER3 polymorphism.

		PER3^4/4^ (n = 9)	PER3^5/5^ (n = 9)	P-value
		Mean (SD) or (%)	Mean (SD) or (%)	
Age (years) *π*		23.89 (2.76)	24.78 (3.35)	0.542
Sex	Male/Female *¥*	1 (11.1%)/8 (89.9%)	2 (22.2%)/7 (78.8%)	0.500
Years of study *π*		15.33 (0.71)	16.33 (1.50)	0.089
Body Mass Index *π*		25.66 (7.21)	22.14 (1.82)	0.175
Pittsburgh Sleep Quality Index *€*		3.56 (2.13)	3.44 (1.24)	0.894
Epworth Sleepiness Scale *€*		9.11 (5.90)	7.44 (2.88)	0.458
Beck Depression Inventory II *€*		6.22 (5.89)	6.67 (3.97)	0.853
State-Trait Anxiety Scale – Trait π		19.44 (3.84)	20.44 (3.05)	0.716
State-Trait Anxiety Scale – State π		21.89 (4.23)	22.56 (3.36)	0.549
**Munich ChronoType Questionnaire**				
MSFsc *π*		3.73 (0.99)	4.10 (0.94)	0.422
Social jetlag *π*		1.43 (0.49)	1.31 (0.70)	0.834
Sleep duration (work days) *π*		7.37 (0.75)	7.25 (1.02)	0.779
Sleep duration (work-free days) *π*		8.81 (0.92)	8.51 (1.22)	0.568

Data are presented as mean (SD) or frequency (%) (total n = 18).

Notes. PER3 = Period 3 (clock) gene; MSFsc = mid-sleep on free days corrected for sleep debt accumulated over the workweek. The statistical test used to compare the groups are identified by the symbols close to the name of variables.

π t-Test for independent samples.

¥ Fisher’s Exact Test.

€ Wilcoxon Mann-Whitney Test.

The HPT, CPM-task, and CPT measurements for the morning and afternoon periods according to genotype groups (PER3^4/4^ *vs*. PER3^5/5^) are presented in Table 2. We used the Wilcoxon-Mann-Whitney test for a dependent sample to assess the temporal patterns of the pain measures and biomarkers of neuroplasticity (BDNF and S100-β). The analysis did not find a statistically significant difference within the PER3^4/4^ or the PER3^5/5^ group for these measures.

**Table 2:** The within comparisons in the pain measures, S-100B, and BDNF on the genotypes groups PER3^4/4^ and PER3^5/5^ (means obtained in the afternoon and morning).

	PER3^4/4^ (n = 9)	∆-value	P-value*	PER3^5/5^ (n = 9)	∆-value	P-value*
	*Mean (SD)*			*Mean (SD)*		
**Heat Pain Threshold (** ^**0**^ **C degree)**						
Morning	43.30 (2.35)	−0.31	0.47	42.53 (3.49)	−0.26	0.86
Afternoon	42.99 (1.62)			42.27 (3.48)		
**Change on Numerical Pain Scale (0–10) during CPM-task**						
Morning	−2.44 (1.42)	−0.41	0.52	−2.78 (0.96)	0.67	0.06
Afternoon	−2.85 (1.30)			−2.11 (0.93)		
**Cold Pressor Test (seconds)**						
Morning	40.33	−0.11	0.89	38.11	−4.00	0.04
Afternoon	40.22			34.11		
**Brain-derived Neural Factor (ng/ml)**						
Morning	22.56 (8.21)	7.69	0.03	30.62 (8.87)	11.80	0.44
Afternoon	30.25 (12.24)			42.42 (23.53)		
**S100-B protein (ng/ml)**						
Morning	24.67 (17.68)	−7.63	0.13	27.50 (14.37)	1.32	0.77
Afternoon	17.04 (8.54)			28.82 (17.82)		

Data are shown as mean and (SD) (n = 18).

Notes. CPM = Conditioned Pain Modulation. CPT = Cold Pressor Test. P-values are based on t-test for independent samples.

*P-value of comparison between delta values (afternoon minus morning) within PER3^4/4^ and PER3^5/5^ by the Wilcox-Mann Whitney test.

We explored the relationships between the pain measures and other variables of interest, namely, chronotype indicators associated with sleep time, S100-β protein and BDNF, regardless of genotype. Spearman correlation analyses are presented in Table 3. A late sleep midpoint (MCTQ MSFsc) was correlated positively with the change in the NPS (0–1) during the CPM-task measured in the afternoon. This indicates that the DPMS tends to be less efficient in the afternoon in subjects with late phase of entrainment. Also, the S100-β protein is negatively correlated with the CPT, independently of the time of measurement. Thus, this relationship suggests that the serum level of the astrocytic protein S100-β was inversely correlated to pain tolerance.

**Table 3:** Spearman correlations (ρ) between pain psychophysics measures, chronotype measures and serum markers of neuroplasticity (n = 18).

	CPM task morning	CPM task afternoon	CPT morning	CPT afternoon
MCTQ MSFsc	0.294	**0.495** ^*^	0.112	0.188
MCTQ Social Jetlag	0.255	0.209	0.185	0.196
BDNF morning	0.116	0.345	0.278	0.126
BDNF afternoon	−0.080	0.258	0.038	−0.035
S100β morning	−0.132	0.270	**−0.536** ^*^	−0.401
S100β afternoon	−0.069	0.110	**−0.516** ^*^	−0.418

Note. CPM = conditioned pain modulation; CPT = Cold Pressor Test; MCTQ = Munich ChronoType Questionnaire; MSFsc = mid-sleep on free days corrected for sleep debt accumulated over the workweek; BDNF = Brain-Derived Neurotrophic Factor. *p < 0.05.

### Primary outcome

#### Analysis of the influence of the PER3^4/4^ and PER3^5/5^ genotypes on the temporal pattern of descending pain modulatory system function

**Figure 3:**
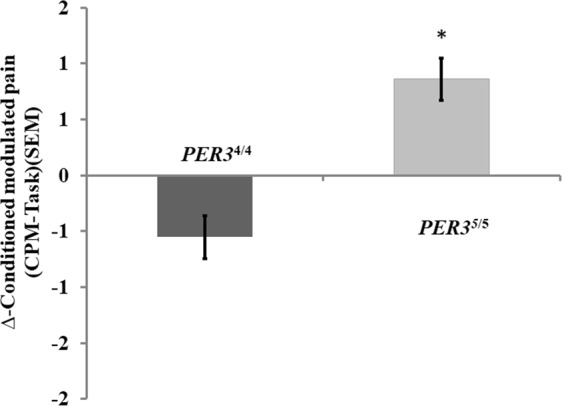
Average of the ∆-CPM of the clock genes groups PER3^4/4^ and PER3^5/5^ compared by a GLM. Errors bars indicate the mean standard error (SEM). Data are presented as mean and (SEM). Asterisks show a significant difference after the adjustment for the Bonferroni Test for multiple comparisons.

The PER3^4/4^ group presented a smaller mean change in the NPS (0–10) during the CPM-task across the day (∆-CPM from afternoon to morning) compared to the PER3^5/5^ group. The mean (SD) ∆-CPM for the PER3^4/4^ and PER3^5/5^ genotypes were −0.41 (0.78) and 0.67 (0.90), respectively. A GLM revealed a statistically significant difference in the ∆-CPM between genotypes PER3^4/4^ and PER3^5/5^ (χ^2^ = 7.26, DF = 1; P = 0.007). The results are presented in Fig. 3.

Considering that sleep deprivation is an important factor related to pain, we included sleep deprivation in the GLM as a covariate. It was correlated negatively with the ∆-CPM. That is, more extended sleep deprivation correlated with a higher value of ∆-CPM, indicating that the descending pain modulatory system was less efficient. No interaction was observed between the effects of the amount of sleep deprivation and the PER3^4/4^ or PER3^5/5^ genotype on the functioning of the DPMS. This result suggests that the impact of sleep deprivation of at least 120 min existed independently of *PER3* VNTR polymorphism (Table 4).

**Table 4:** Primary outcome – generalized linear model analyses to compare the ∆-CPM between genes groups PER3^4/4^ and PER3^5/5^.

Parameter	B	SE	CI 95%	χ^2^	df	P-value	Effect size
(Intercept)	1.778	0.6438	(0.51 to 3.04)	7.627	1	0.006	
Gene PER3^4/4^	−2.446	0.9575	(−4.32 to −0.57)	6.528	1	0.011	0.64
Gene PER3^5/5^	0^a reference^						
Sleep deprivation lower than 2 (h)	−0.969	0.4432	(−1.84 to −0.11)	4.785	1	0.029	
∆- S100β- protein	−0.030	0.129	(−0.06 to −0.02)	5.475	1	0.019	0.55
∆- BDNF	0.015	0.105	(0.01 to 0.03)	3.905	1	0.048	
Gene PER3^4/4^*Sleep deprivation (h)	0.907	0.6509	(−0.37 to 2.18)	1.941	1	0.164	
Gene PER3^5/5^*Sleep deprivation (h)	0^a reference^						
(Scale)	0.364^b^	0.1214	(0.18 to 0.70)				

Data were present as mean (SD) (n = 18).

∆- S100-β (from afternoon minus morning). Sleep deprivation was estimated by the social jet lag.

Df = degrees of freedom; *P < 0.05 indicates significant differences between treatment in the estimated marginal means adjusted for multiple comparisons by Bonferroni test.

χ^2^, Wald Chi-Square, CI, confidence interval; B, regression coefficient; SE, standard error.

The Cramer’s V was used as a measure of effect size for qui-square tests. The size effect was interpreted as follows: Standards for interpreting Cramer’s V as proposed by Cohen (1988) are the following: DF (degrees of freedom) = 1 (0.10 = small effect) (0.30 = medium effect) (0.50 = large effect).

https://www.campbellcollaboration.org/escalc/html/EffectSizeCalculator-R5.php.

### Secondary outcomes

#### Relationships between psychophysical pain measures, BDNF, S100-β protein, sleep deprivation and PER3 genotypes

**Figure 4:**
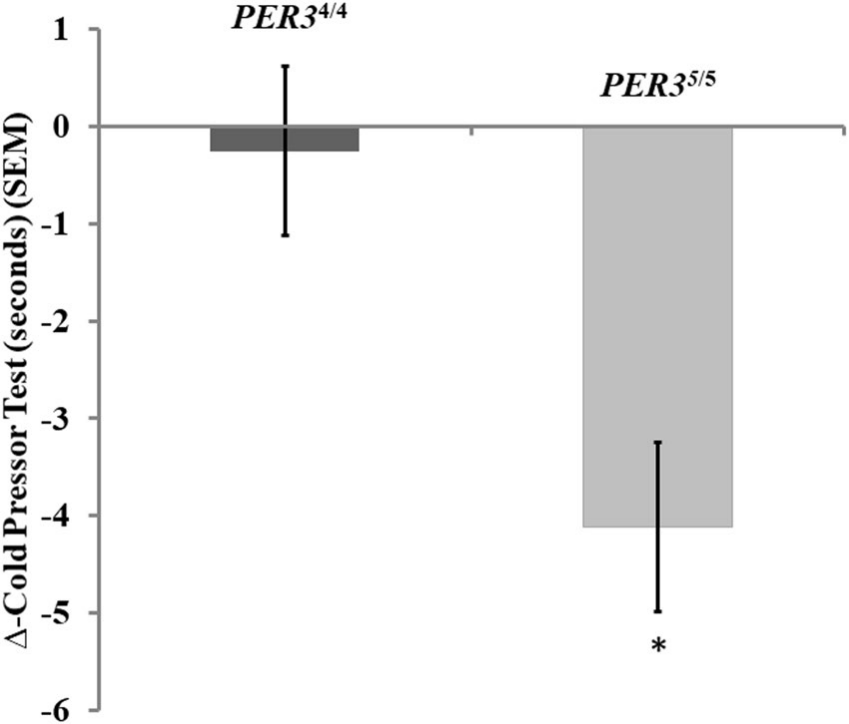
Average of the ∆-CPT of the clock genes groups PER3^4/4^ and PER3^5/5^ compared by a GLM. Errors bars indicate the mean standard error (SEM). Data are presented as mean and (SEM). Asterisks show a significant difference after the adjustment for the Bonferroni Test for multiple comparisons.

The PER3^4/4^ group presented a minimal reduction in the CPT across the day (∆-CPT from afternoon to morning) compared to the PER3^5/5^ group. The mean (SD) ∆-CPT values for the PER3^4/4^ and PER3^5/5^genotypes were −0.40 (2.51) and −4.11 (2.6), respectively. A GLM revealed a statistically significant difference in the ∆-CPT between the PER3^4/4^ and PER3^5/5^ genotypes (χ^2^ = 22.25, DF = 1; P < 0.001). This finding suggests that PER3^4/4^ homozygotes presented smaller changes in the response to painful heterotopic stimulus across the day. These results are summarized in Fig. 4.

The covariates, ∆-S100-β protein, ∆-BDNF and sleep deprivation, were not correlated with the ∆-CPT. There was, however, an interaction between sleep deprivation and genotype. The reduction in tolerance of an intense nociceptive stimulus induced by the immersion of the hand in cold water (0 to 1 °C) existed independently of sleep deprivation (Table 5). However, the interaction analysis showed that the difference between genotype groups PER3^4/4^ and PER3^5/5^ persisted, but that sleep deprivation explains the inversion in the difference in the ∆-CPT across the day. That is, sleep deprivation explains the larger increase in ∆-CPT observed in the PER3^5/5^ group compared to the PER3^4/4^ group.

**Table 5:** Secondary outcome – generalized linear model analyses to compare the ∆-CPT according to PER3 polymorphism genotypes.

Parameter	B	SE	CI 95%	χ^2^	df	P-value
(Intercept)	−7.082	2.0179	(−11.03 to −3.12)	12.31	1	<0.001
Gene PER3^4/4^	15.135	3.2086	(8.85 to 21.42)	22.25	1	<0.001
Gene PER3^5/5^	0^a reference^					
Sleep deprivation (h)	2.312	1.3745	(−0.38 to 5.00)	2.82	1	0.093
∆- S100β	0.012	0.0526	(−0.09 to 0.11)	0.05	1	0.817
Gene PER3^4/4^* Sleep deprivation (h)	−8.652	2.2450	(−13.05 to −4.25)	14.85	1	<0.001
Gene PER3^5/5^* Sleep deprivation (h)	0^a reference^					
(Scale)	6.525b	2.1751	(3.39 to 12.54)			

Data were present as mean (SD) (n = 18).

∆- S100β (from afternoon minus morning).

Df = degrees of freedom; *P < 0.05 indicates significant differences between treatment in the estimated marginal means adjusted for multiple comparisons by Bonferroni test.

χ^2^, Wald Chi-Square, CI, confidence interval; B, regression coefficient; SE, standard error.

## Discussion

This study confirmed the hypothesis that there is a circadian pattern in the inhibitory potency of the DPMS according to the *PER3* VNTR polymorphism. The more substantial change in the ∆-CPM-task across the day occurred in the group with the PER3^5/5^ genotype. This result indicates that this group, which has a delayed sleep phase, presented a lower inhibitory potency in the afternoon. The finding related to CPT is similar, since the ∆-CPT in the PER3^5/5^ genotype again presented greater change across the day with lower pain tolerance in the afternoon. Also, the difference in the ∆-CPM-task assessed by the NPS (0–10) was negatively correlated with the ∆-S100-β protein, while it was positively correlated with the serum ∆-BDNF.

Although our findings do not allow us to test predictions of polymorphism in the PER3 gene as downstream pathways regulated by the molecular clock, it is likely that the different genotypes (PER3^4/4^ and PER3^5/5^) may change neuroplasticity properties, which would explain the different responses in the circadian variation of psychophysical pain measures, nominally CPT and CPM-task. These results showed that top-down pain inhibition during the CPM-task changed in opposite directions across the day in the two groups. While in the PER3^4/4^ homozygotes, the inhibitory function of the DPMS increases from morning to afternoon, in the PER3^5/5^ homozygotes, it decreases. The importance of these results is to show the relative impact of the polymorphism of the *PER3* gene as a mechanistic explanation for the relationships observed between PER3 genotypes and circadian changes in pain processing. Also, we found that the relationship between disinhibition in the DPMS and sleep deprivation is independent of PER3 polymorphism. Although our findings do not allow testing predictions about downstream molecular pathways regulated by the molecular clock, they can help to comprehend the relationships of PER3 polymorphisms with circadian typology, sleep deprivation and the inhibitory function of the DPMS. Although delayed sleep phase subjects can be prone to sleep deprivation, our findings suggest that the PER3 polymorphisms and sleep deprivation can influence the inhibitory potency of the DPMS independently. Although the mechanism that underpins this association is not entirely comprehended, earlier studies suggest that the PER3 polymorphisms, and perhaps sleep deprivation, can cause downstream physiological changes in pain processing, which may be dependent on and/or independent of the central circadian clock. This hypothesis is plausible since the frequency of PER3^5/5^ has been linked to mood disorders in some studies^37–39^ as well as to proneness to anxiety^9^. Likewise, sleep deprivation can alter the central systems involved in pain processing in the spinal cord by decreasing the inhibitory activities of the DPMS and by increasing descending pain facilitatory activity^12,27^. Thus, the novelty of these results is that they provide additional data about the influence of the PER3 polymorphisms on the DMPS. One candidate explanation for these results is imbalance in the neurotransmission of the systems that comprise the DPMS (i.e., noradrenergic, GABA-ergic, glutamatergic (mGLUR5, NMDA), and nitric oxide synthase)^40–42^.

The negative correlation between the S100-β protein and the CPT, as well the relationship of S100-β with the change in the CPM-task across the day, suggests an interplay between the astrocytic-glial activity indexed by the S100-β protein and the DPMS. These findings are aligned with our previous study of fibromyalgia, where we found an inverse association between serum S100-β protein levels and the pain pressure threshold^25^. Although the mechanism underlying this association is unclear, the results of an experimental study have demonstrated that there is a spinal cord circadian expression of the clock genes that is dependent on the activity of astrocytes^43^. As the S100-β level represents white matter structural changes, it might be a surrogate marker of neuroglial activity. Hence, this finding can help to comprehend the role of neuroglia and their interplay with the DPMS^24^. However, this is a study with human subjects, so we cannot isolate the effect of each system to define the specific role of each marker of neural plasticity.

In the current study, no significant difference was observed in the ∆-HPT across the day between the PER3^4/4^ and PER3^5/5^ genotypes (see Table 2). It is possible that under a minimal stimulus, the influence of the homeostatic change in pain processing could be less evident. Whereas, in severe acute pain, intense nociceptive stimulus (CPM-task, CPT) or chronic pain, the impacts of homeostatic change in the ∆-CPM-task on top-down inhibition could be more robust. This interpretation has biological plausibility from experimental and clinical studies^44–48^. For example, PER2 mutant mice continued to exhibit daily rhythms in arthritic inflammatory pain^48^. Eight to twelve-hour shifts can occur in inflammatory and neuropathic pain in the daily rhythms of pain sensitivity^44–47^. Thus, the present findings highlight an influence of homeostatic mechanisms of inhibition in the DPMS across the day and in a top-down manner according to genotypic (PER3^4/4^ and PER3^5/5^) and phenotypic (MCTQ-MSFsc) characteristics.

We did not observe a relationship between BDNF variation across the day and the PER3^4/4^ and PER3^5/5^ genotypes. However, ∆-BDNF is associated with the change in the CPM-task across the day. This relationship is substantiated by a set of previous studies that showed the effects of BDNF on the neuroplasticity of the DPSM. The positive correlation of BDNF with the change in the DPMS across the day can be an additional reason why it has been suggested as a marker of the central sensitization process in chronic pain conditions^23,49^. Although many studies have shown a circadian rhythm in BDNF secretion^50,51^, according to the results of this study, its secretion across the day is not related to the PER3 polymorphisms. In the interpretation of this effect, we need to take into account that the circulating BDNF represents approximately 70–80% of the BDNF produced in the central nervous system, but it is transported through the blood-brain barrier via saturable systems^52,53^. Thus, additional studies with measures in loco or with knockout animals are needed to allow definitive conclusions about the relationship between the PER3^4/4^ and PER3^5/5^ genotypes, the rhythm of BDNF secretion across the day, and the change in the CPM-task across the day.

We should interpret these findings parsimoniously, because the changes in the DPMS can be linked indirectly to the clock genes and biomarkers of neuroplasticity (i.e., BDNF and S100-β). Although the design of this study prevents determining a cause-consequence relationship, it does permit us to better understand the temporal pattern of variation of the DPMS. Also, one needs to consider that the perception of pain can be affected by several factors, such as previous experiences^54^, gonadal hormones, sex, ethnicity, and personality traits^55,56^. Thus, it is essential to differentiate the variations caused by these characteristics from those produced by the circadian fluctuations triggered by different chronotypes or clock genes. In our research, the evaluations were performed in a constant environment to attempt to control this bias, as we maintained the same evaluation room with controlled temperature, an unvarying assessment protocol according to guidelines, and the same researchers to apply all tests. Nevertheless, future studies are required to determine how the *PER3* VNTR polymorphism may be responsible for the change in the DPMS in both acute and chronic pain. Additionally, further studies are needed to assess whether this polymorphism may be a useful predictor for identifying subjects prone to chronic pain.

These results provide additional evidence to bring attention to the influence of the PER3^4/4^ and PER3^5/5^ genotypes on the efficiency of the DPMS. Also, they suggest that the secretions of BNDF and S100-β protein modulate the change in the DPMS across the day. Sleep deprivation was found to be an independent factor affecting the efficiency of the DPMS, despite the effect of the PER3 polymorphism. Overall, these findings might be useful for comprehending the potential impact of *PER3* VNTR polymorphism as targets to improve DPMS function, which can facilitate chronotherapeutic advances in pain treatment and serve as a potential predictor to individualize the pain treatment.
